# Influence of Menisci on Tibiofemoral Contact Mechanics in Human Knees: A Systematic Review

**DOI:** 10.3389/fbioe.2021.765596

**Published:** 2021-12-03

**Authors:** Matthias Sukopp, Florian Schall, Steffen P. Hacker, Anita Ignatius, Lutz Dürselen, Andreas M. Seitz

**Affiliations:** Institute of Orthopaedic Research and Biomechanics, Center of Trauma Research Ulm, Ulm University Medical Center, Ulm, Germany

**Keywords:** tibiofemoral contact, contact pressure, systematic review, meniscus injuries, osteoarthritis, PTOA

## Abstract

**Purpose:** Menisci transfer axial loads, while increasing the load-bearing tibiofemoral contact area and decreasing tibiofemoral contact pressure (CP). Numerous clinical and experimental studies agree that an increased CP is one predominant indicator for post-traumatic osteoarthritis (PTOA) of the knee joint. However, due to the immense variability in experimental test setups and wide range of treatment possibilities in meniscus surgery, it is difficult to objectively assess their impact on the CP determination, which is clearly crucial for knee joint health. Therefore, the aim of this systematic review is to investigate the influence of different meniscal injuries and their associated surgical treatments on the CP. Secondly, the influence of different test setups on CP measurements is assessed. On the basis of these results, we established the basis for recommendations for future investigations with the aim to determine CPs under different meniscal states.

**Methods:** This review was conducted in accordance with the PRISMA guidelines. Studies were identified through a systematic literature search in Cochrane, PubMed and Web of Science databases. Literature was searched through pre-defined keywords and medical subject headings.

**Results:** This review indicates a significant increase of up to 235% in peak CP when comparing healthy joints and intact menisci with impaired knee joints, injured or resected menisci. In addition, different test setups were indicated to have major influences on CP: The variety of test setups ranged from standard material testing machines, including customized setups *via* horizontal and vertical knee joint simulators, through to robotic systems. Differences in applied axial knee joint loads ranged from 0 N up to 2,700 N and resulted unsurprisingly in significantly different peak CPs of between 0.1 and 12.06 MPa.

**Conclusion:** It was shown that untreated traumatic meniscal tears result in an increased CP. Surgical repair intervention were able to restore the CP comparable to the healthy, native condition. Test setup differences and particularly axial joint loading variability also led to major CP differences. In conclusion, when focusing on CP measurements in the knee joint, transparent and traceable *in vitro* testing conditions are essential to allow researchers to make a direct comparison between future biomechanical investigations.

## Introduction

Traumatic meniscus injuries are one of the most predominant risk factors for post-traumatic osteoarthritis (PTOA) (Cooper et al., 2000; Wilder et al., 2002; Roos, 2005), resulting in a considerable socioeconomic burden globally (Felson and Zhang, 1998). The semilunar, fibrocartilaginous knee joint menisci play a crucial role in load-bearing and load transmission within the knee joint (Walker and Erkman, 1975; Fukubayashi and Kurosawa, 1980). During knee joint movements, the wedge-shaped menisci actively increase the load-bearing contact area by compensating the incongruency of the articular surfaces of the tibia and femur, resulting in a decreased tibiofemoral contact pressure (CP) (Walker and Erkman, 1975).

A typical meniscus injury mechanism is external tibial rotation in combination with axial loading during knee flexion (Nielsen and Yde, 1991), which frequently occurs during sports activities like football, basketball, soccer, and skiing (Baker et al., 1985). Depending on the injury mechanism, various types of meniscal tears can occur and they are categorized in accordance to their location and shape. In the outer third zone—the so-called vascularized, “red” zone—the gold standard of meniscal tear treatment is suturing (Rankin et al., 2002; Makris et al., 2011), while repair in the avascular, “white” zone is commonly not indicated, because of a poor healing potential. Therefore, tears that are localized in this avascular zone are predominantly treated by (partial) meniscectomy (Makris et al., 2011) or being replaced using different substitutes (Linke et al., 2006; de Groot, 2010; Stein et al., 2019). In more severe cases, like permeating root or radial tears, which are described to be biomechanically equivalent to a total meniscectomy, the major function of the meniscus is completely lost (Allaire et al., 2008). In such cases, the CP is dramatically increased, which will lead, when untreated, in the long term to the development of premature knee joint PTOA (Felson et al., 2000; Heckelsmiller et al., 2017).

**FIGURE 1:**
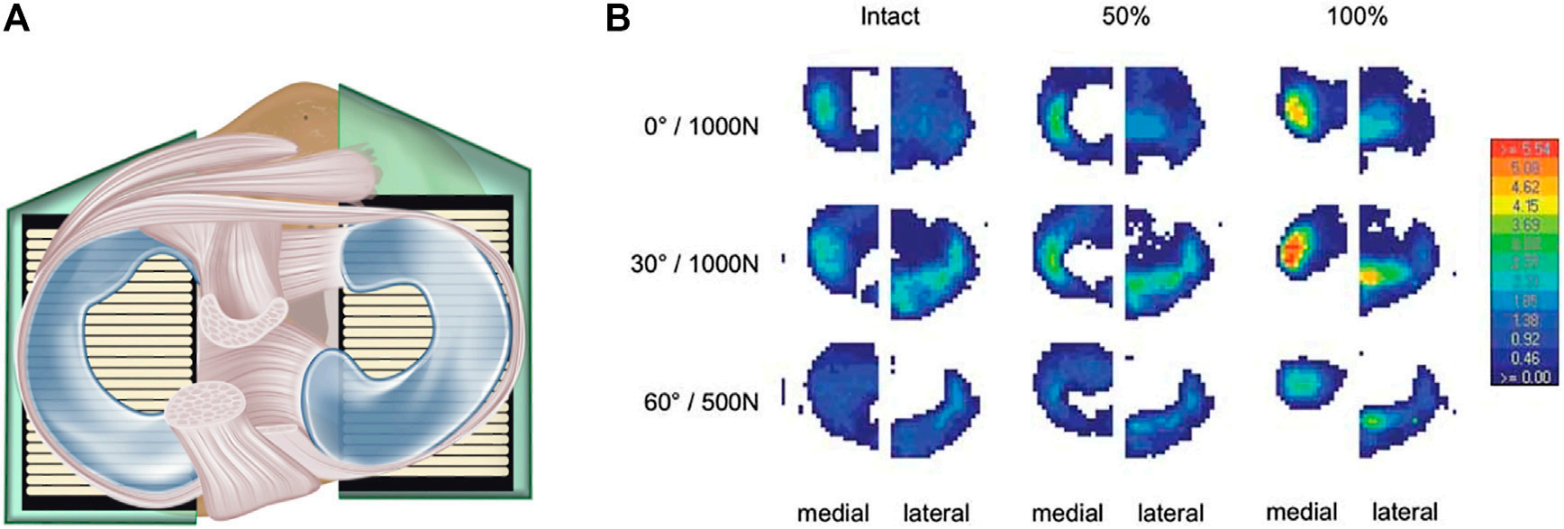
**(A)** Axial view. Schematic drawing of a knee joint with intact menisci, including an equipped pressure-sensitive foil sensor between the menisci and the tibia to measure tibiofemoral contact mechanics *in vitro*. The femur is removed to allow better visibility of the sensor placement inside the knee. **(B)** Example of a tibiofemoral contact measurement: “Contact pressure at the tibial plateau of a representative knee. The pattern of the sensors shows the lateral and medial compartments under intact, 50 and 100% partial resection state at two flexion angles (0 and 30°) under an axial load of 1,000 N and at 60° flexion under 500 N, respectively. The anterior aspect of the joint is pointing upwards. The legend on the right side indicates the pressure in MPa.” Reprinted with permission from Seitz et al. (2019).

Experimentally, the CP is normally determined during *in vitro* experiments use a large variety of test setups, ranging from customized setups that are integrated in a standard material testing machine, horizontal, and vertical rigs (Oxford-rig) and robotic systems. The knee joint motion during the tests is achieved either *via* an actuator (passive movement) or actively by simulating muscle forces, for example, the quadriceps muscle for extension. In most investigations, the CP is measured between the menisci and the tibial plateau by means of a pressure sensitive film (Figure 1). However, due to the immense variability in experimental test setups, including the respective loading regimes, and wide range of treatment possibilities in meniscus surgery, it is sometimes difficult to objectively assess their mutable impact on the CP determination, which is clearly crucial for knee joint health. Therefore, the main aim of the present systematic review was to investigate the influence of the intact, injured, repaired, or resected meniscal state on the CP. Secondly, we evaluated the impact of different test setups and conditions—for example, variabilities in axial force, knee alignment and muscle simulation on the CP. This overview of currently available testing conditions, which have been used to investigate the impact of different meniscal states on CP measurements, allowed us to establish the basis for recommendations that allow for transparent and reproducible test conditions for future biomechanical CP studies.

## Methods

### Search Strategy

This review was conducted in accordance with the PRISMA guidelines (Moher et al., 2009). A comprehensive and systematic review of the literature was performed to identify studies investigating the meniscal influence on the tibiofemoral contact mechanics in human knee joints. For the literature research, the Cochrane, PubMed and Web of Science databases were used. The literature search strategy was developed using a combination of keywords and medical subject heading (MeSH; Table 1), which were extended to maximize the inclusion of potentially relevant studies.

**TABLE 1:** Queries and search results for Cochrane, PubMed and Web of Science with the number of publications found.

Database	Query	Items found
Cochrane-PubMed-Web of Science	(knee*) AND (contact*) AND (mechanic*) AND (menisc*) AND (pressure)	93
	(knee*) AND (analysis*) AND (pressure*)	1,495
	(knee*) AND (contact pressure*) AND (tibiofemoral*)	174
	(knee joint*) AND (biomechanics*) AND (contact*)	604
	(knee joint*) AND (contact*) AND (pressure*)	640
	(knee*) AND (contact mechanism*)	428
	MeSH: (knee*) AND MeSH: (contact*) AND MeSH: (mechanic*) AND MeSH: (menisc*) AND MeSH: (pressure*)	125

### Study Selection

The results of the database searches were transferred into Endnote (Clarivate Analytics, PA, United States), where the references were automatically updated and duplicates removed. All titles and abstracts of the identified publications were screened. Papers containing *in vitro* tibiofemoral CP data and those investigating healthy, non-degenerated menisci, meniscal interventions, meniscal tears, repair or replacement or meniscectomy and their influence on the CP were included. Per requirement, the CP must also be clearly assignable to the respectively used knee flexion angles (0, 30, and 60°). Studies with computational/simulation approaches, studies in which the focus was other than on human knee joints, gender impact studies and those investigating knees with pathological alterations were excluded. Additionally, the reference lists of the selected publications were screened to include relevant studies that were missed during the previously described selection process.

**FIGURE 2:**
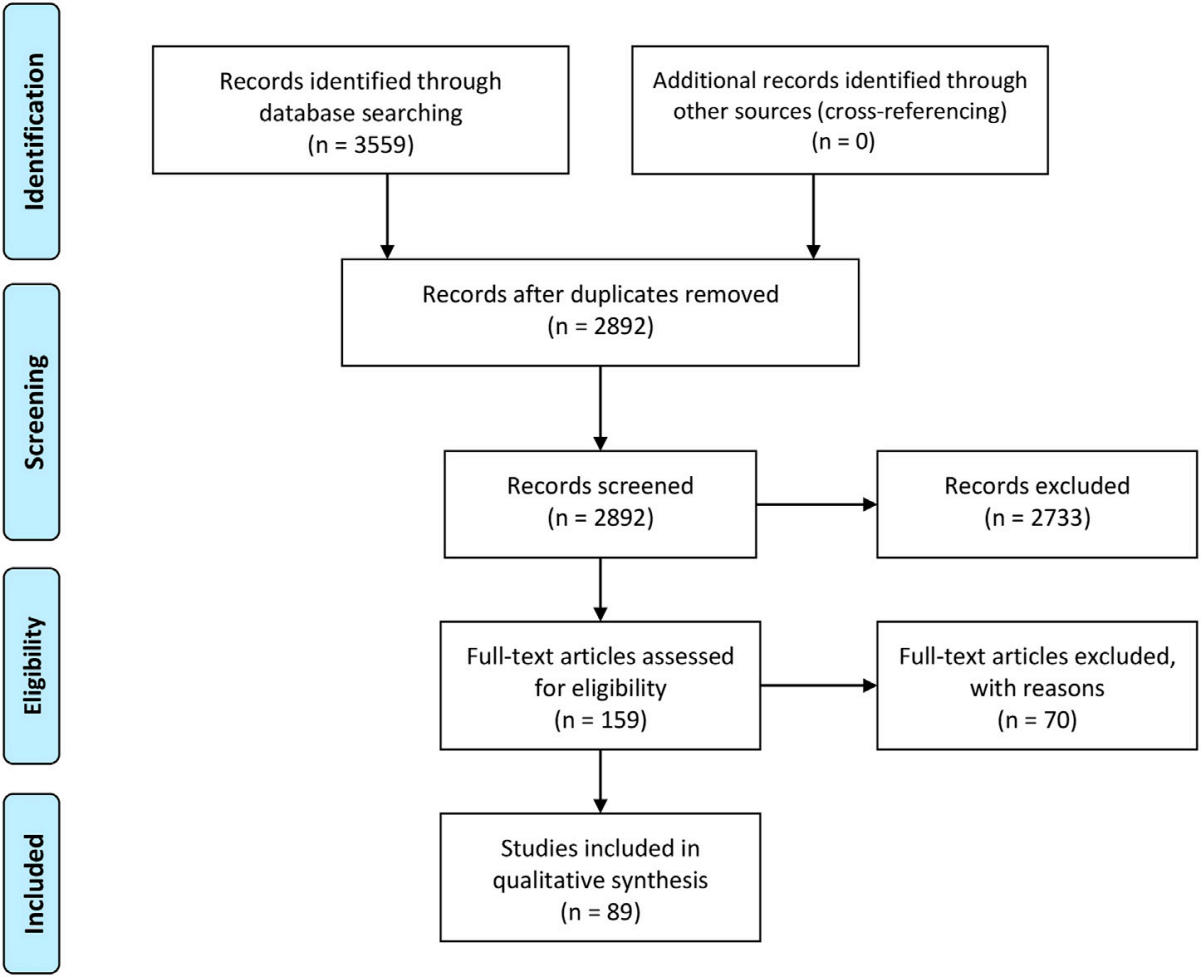
Flow diagram of the study selection process; according to the PRISMA guideline, last update: 2021/08/01.

All publications that included CPs were evaluated graphically, indicating axial loads, meniscal states, interventions, knee joint compartment and the respective peak CP. Only studies directly reporting CP values were investigated and analyzed. The detailed selection process of the study selection is given in Figure 2. The full text of the remaining articles was then analyzed and data from eligible studies were extracted.

## Results

**FIGURE 3:**
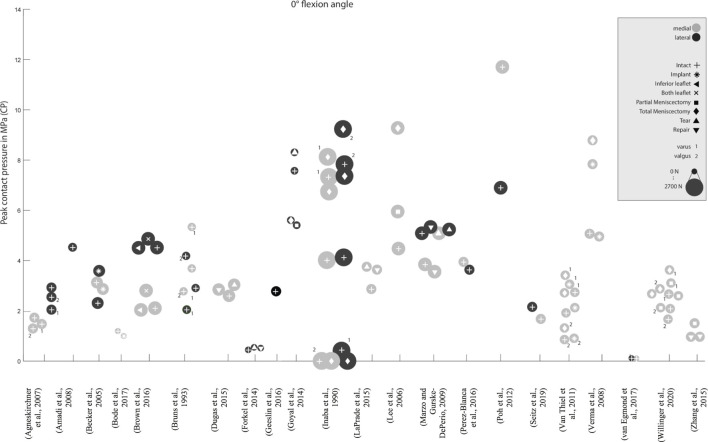
Peak contact pressure at 0° knee joint flexion. Differentiation between lateral, medial and meniscal states. Data point size represents the magnitude of the applied axial load ranging from 0 N to 2,700 N. (Data extracted from written values).

**FIGURE 4:**
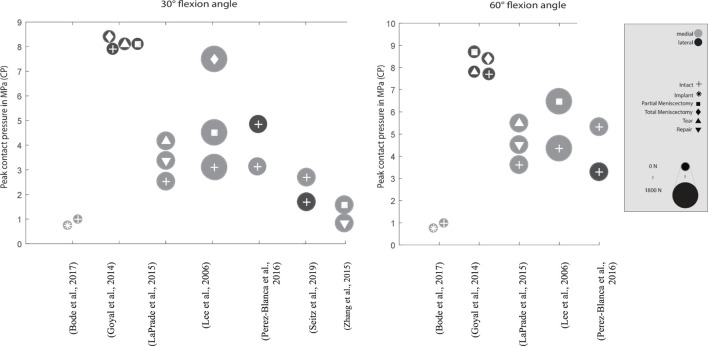
Peak contact pressure at 30° and 60° knee joint flexion. Differentiation between lateral, medial and meniscal states. Data point size represents the magnitude of the applied axial load ranging from 0 N to 1,800 N. (Data extracted from written values)

**FIGURE 5:**
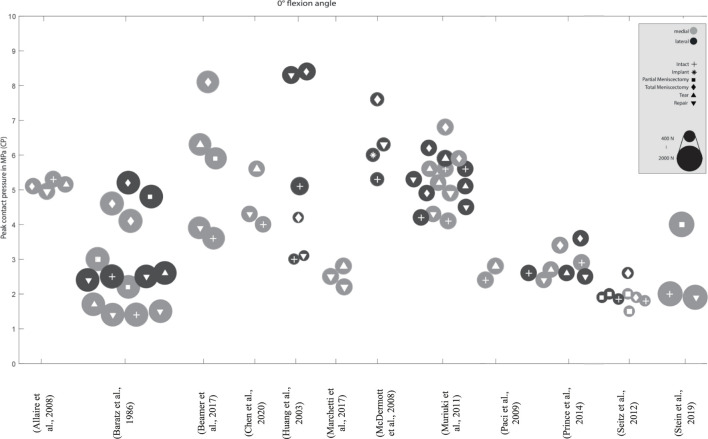
Peak contact pressure at 0° knee joint flexion. Differentiation between lateral, medial menisci and meniscal states. Data point size represents the magnitude of the applied axial load ranging from 400 N to 2,000 N. (Data approximated from graphs).

**FIGURE 6:**
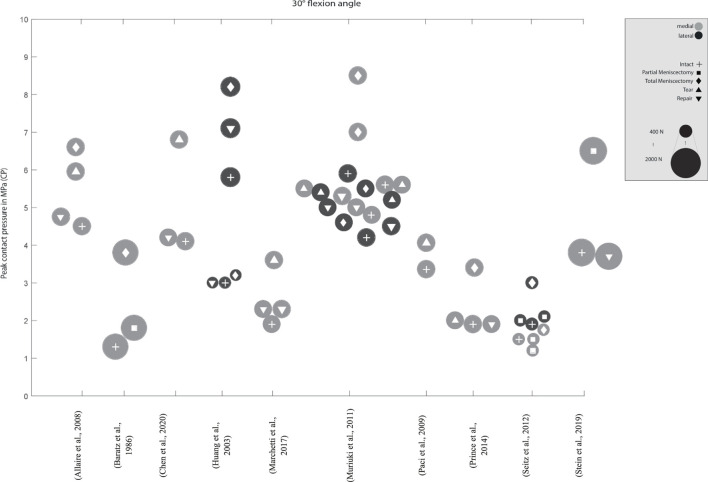
Peak contact pressure at 30° knee joint flexion. Differentiation between lateral, medial menisci and meniscal states. Data point size represents the magnitude of the applied axial load ranging from 400 N to 2,000 N. (Data approximated from graphs).

**FIGURE 7:**
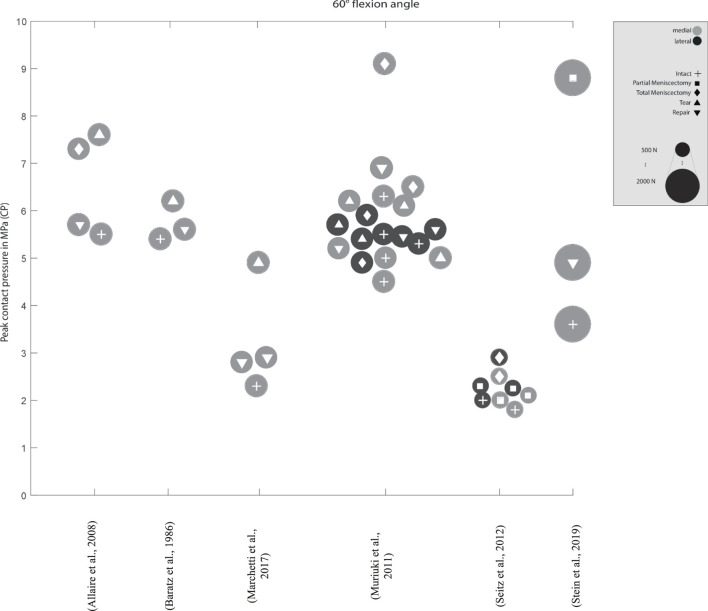
Peak contact pressure at 60° knee joint flexion. Differentiation between lateral, medial menisci and meniscal states. Data point size represents the magnitude of the applied axial load ranging from 500 N to 2,000 N. (Data approximated from graphs).

Following removal of duplicates and exclusions made based on the exclusion criteria, the strategic search resulted in 89 publications (Figure 2; Supplementary Material). Of these, 34 publications were additionally evaluated graphically. These publications are listed in more detail in the diagrams below (Figures 3–7). The remaining 55 publications could not be graphed because no graphing information was provided. Nonetheless, they were evaluated in the text in the following chapters. The following subdivisions were used for detailed comparisons: *Meniscal states, test setups, loading application* and *muscle force simulation*.

### Meniscal states

**FIGURE 8:**
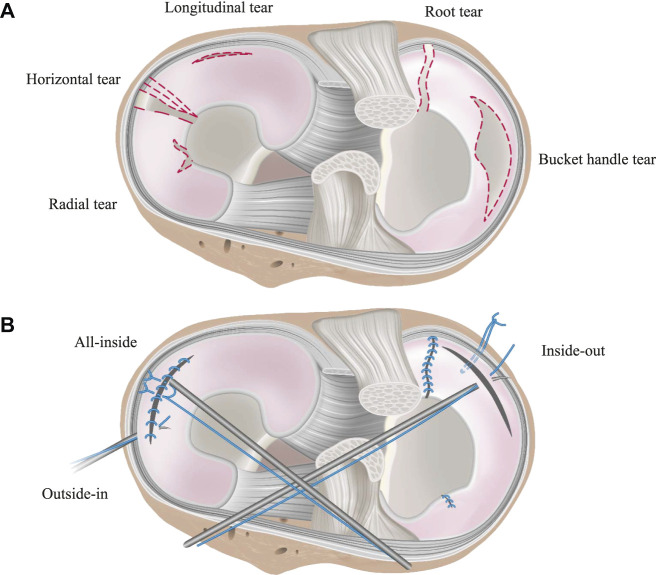
Schematic illustration of the tibial plateau, representing **(A)** traumatic and damaged meniscal states and **(B)** repaired meniscal state. Meniscal tears shown in **(A)** are the most common tears, presented widely in the literature. Suture techniques for their treatment are shown in **(B)**.

**FIGURE 9:**
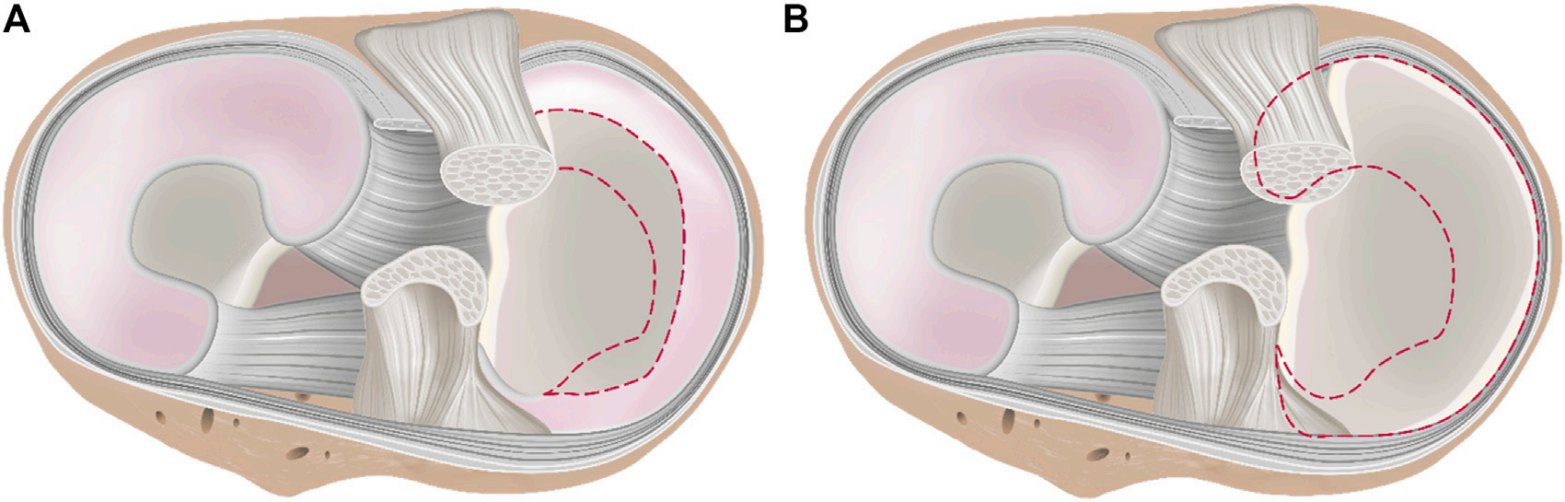
Schematic illustration of the tibial plateau with **(A)** partial meniscectomy and **(B)** total meniscectomy. The dashed line indictes the removed meniscus tissue in each case.

From a biomechanical point of view, a reduced tibiofemoral contact area caused by meniscal tears (Figure 8) or partial or total meniscectomy (Figure 9) can potentially lead to premature gonarthrosis. Therefore, we investigated the effects of meniscal injuries and their surgical treatments on the tibiofemoral CP in this chapter.

#### Meniscal Tears

Forkel et al. found, that an isolated root tear of the lateral meniscus led to an increased peak CP by 23% compared to the intact meniscal state, while a meniscus root tear with an associated transection of the meniscofemoral ligament led to an increase of 225% compared to the intact state. Furthermore, they showed that an anatomic transtibial fixation of the tear or alternatively with a transtibial fixation *via* an anterior cruciate ligament tunnel restores the CP values in the lateral compartment to that comparable to the intact state (Forkel et al., 2014). Schillhammer et al. measured a 50% increase in CP in the case of a posterior horn detachment (Schillhammer et al., 2012). Furthermore, the authors showed that the peak CP in the lateral compartment can be reduced to the intact level by means of a transtibial tunnel repair (Schillhammer et al., 2012). Marzo and others investigated a 32% increase in CP in the medial compartment after simulating a posterior root tear of the medial meniscus (Marzo and Gurske-Deperio, 2009). They concluded, that such a tear can cause the meniscus to extrude out of the joint, leading to a loss of the ability to absorb hoop stresses, finally resulting in an increased peak CP (Allaire et al., 2008; Marzo and Gurske-Deperio, 2009). Repair of the meniscus after a posterior horn tear of the medial meniscus with a transosseous suture (Marzo and Kumar, 2007) restored the hoop stress resistance of the meniscus with a peak CP similar to healthy knee joints (Allaire et al., 2008; Marzo and Gurske-Deperio, 2009). Furthermore, Chen et al. (1996) and Paletta et al. (1997) corroborated these results. Therefore, it can be concluded that a transtibial suture repair of a posterior meniscus root tear is able to restore the CP to that comparable to the intact meniscus situation.

Zhang et al. investigated the treatment of a radial meniscus tear in the medial meniscus with an all-inside meniscal repair technique, an inside-out repair and a partial meniscectomy on the peak CP (Zhang et al., 2015). Both, all-inside meniscal repair and the inside-out repair decreased the peak CP by approximately 40% compared to the partial meniscectomy state. In other studies, Bedi et al. (2012) and Padalecki et al. (2014) showed an increase in the medial compartment peak CP proportional to the width of the radial posterior horn tear of the medial meniscus. Lee et al. investigated the outcome on the peak CP after undergoing five consecutive medial meniscectomy conditions, beginning from intact over 50–75% to a segmental and finally total meniscectomy, finding that the medial compartment peak pressures continuously increased from approximately 4.5–9.27 MPa at 0° flexion angle, respectively (Lee et al., 2006). At a deflection angle of 30°, even an increase of 142% was determined. A similar study by Seitz et al. investigated the effect of partial meniscectomies of the medial posterion horn. Here, an increase in CP was reported, although only significantly in higher flexion angles (>30°) of the knee. Total meniscectomy, however, resulted in a significant increase in pressure in all knee flexion angles (Seitz et al., 2012).

Goyal et al. found only a minor increase in the peak CP from subsequent vertical tears in the periphery of the lateral meniscus compared to the intact meniscal state (Goyal et al., 2014). They argued that with the circumferential fibers being still intact in such a tear configuration, the torn meniscus is still able to provide a sufficient load transmission. Chen et al. also compared the medial peak CP of a repaired vertical longitudinal tear with that of the intact and injuried ones (Chen et al., 2020). They reported a significant improvement of tibiofemoral contact conditions after meniscus repair, although the peak CP and area after repair were not significantly different from those of the tear conditions. However, they found at high flexion of over 60°, new cutting effects appearing on the repaired meniscus, which might result in tear gapping and thereby causing new tears.

#### Partial and Total Meniscectomy

Various studies showed a significant 50–200% increase in the peak CP in medial meniscectomized versus intact knees (Fukubayashi and Kurosawa, 1980; Kurosawa et al., 1980; Baratz et al., 1986; Chen et al., 1996; McDermott et al., 2008; Muriuki et al., 2011; Prince et al., 2014). In their study, Brown et al. demonstrated, that after a horizontal medial meniscal tear, the resection of the horizontal inferior leaflet did not alter the peak CP compared to the intact state (Brown et al., 2016). They explained, that in this tear configuration the continued dissipation of hoop stresses is still possible, thereby providing sufficient load transformation from axial loading into circumferrential hoop stresses. However, the resection of both leaflets increased the peak CP in the medial compartment by approximately 35% in comparison to the intact state. They concluded that the resection of both leaflets had a biomechanically dramatic effect through contact area reduction on the articular surfaces. Therefore, axial loading forces can no longer be adequately dissipated from the cartilage surface (Yim et al., 2013), leading to an increased risk for OA (Brown et al., 2016). These findings are supported by the results of Beamer et al., who indicated a moderate 8% increased peak CP after a horizontal cleavage tear repair when compared to the intact meniscal state (Beamer et al., 2017). The post-injured peak CP increase was approximately 70% after a horizontal cleavage tear. Goyal et al. reported an increase in the peak CP after subtotal meniscectomy of the lateral meniscus of up to approximately 12% compared to the intact state, with more pronounced effects during deep knee flexion at 30 and 60° flexion (Goyal et al., 2014). Biomechanically, the meniscal rim is the structure that is most crucial for transferring axial loads into circumferrential hoop stresses and retaining them. Therefore, the increase in the peak CP is dramtically higher when the meniscal rim is involved in a meniscal injury. Zhang et al. found a significant increase in the medial peak CP by simulating a radial tear at the posterior horn of the medial meniscus and a consecutive partial meniscectomy treatment compared to the intact state, indicating a peak CP increase of 67% after radial tear simulation and a 118% increase after partial meniscectomy compared to the intact state (Zhang et al., 2015). Therefore, whenever possible, meniscus tears should be repaired not only to avoid increased CP but also to prevent tear propagation (Goyal et al., 2014).

#### Implants, Partial and Total Meniscus Replacement

Becker et al. investigated the meniscofemoral peak CP in the medial and lateral compartments at full knee extension under an axial load of 1,400 N with five different biodegradable implants for meniscal repair after simulating a bucket-handle tear (Becker et al., 2005). They showed, that the repaired menisci led to a similar peak CP compared to intact knees. In the initial intact state, the medial peak CP was 3.12 MPa and the lateral pressure 2.3 MPa. In the medial compartment of the five implants, the peak CP ranged from 2.66 to 3.74 MPa. Laterally, pressure values between 2.84 and 3.73 MPa were identified.

Stein et al. investigated the use of a biocompatible silk fibroin meniscal replacement (Stein et al., 2019). A partial meniscectomy, leaving a 4 mm peripheral meniscal rim and both meniscal horns intact, was performed and replaced by the implant. Initially, a doubling of the peak CP was observed after partial meniscectomy followed by a restoration of the peak CP to the intact situation after partial replacement. With a flexion angle of 60°, an increase of approximately 140% could be calculated. Subsequently, the initial intact peak CP could be achieved by using replacements (Wang et al., 2015; Stein et al., 2019). Huang et al. examined how different levels of compressive load (400 vs. 1,200 N) affect the CP outcomes after autograft procedures (Huang et al., 2003). The most important result was that an autograft can restore the initial intact peak CP at 400 N axial load. However, at 1,200 N, the restored peak CP were significantly higher than in initial intact state. They concluded that differences, therefore, cannot be scaled up from lower load scenarios to higher ones (Huang et al., 2003).

### Test Setups

**FIGURE 10:**
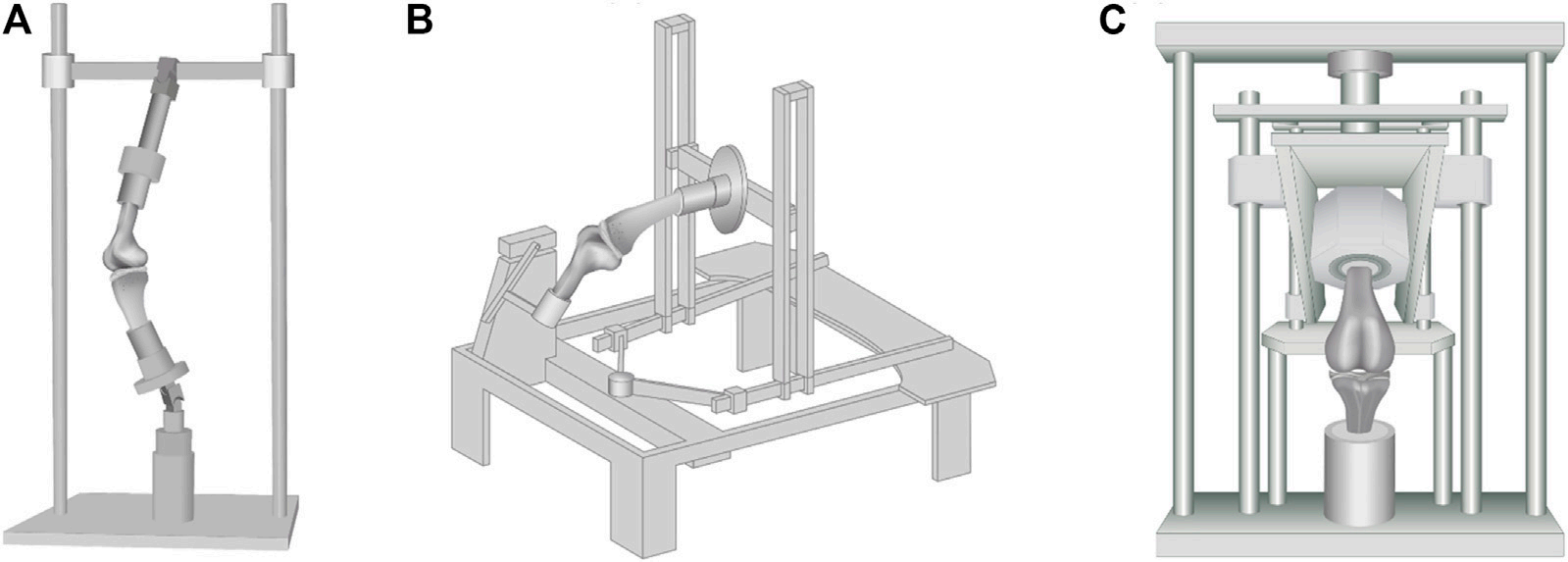
Commonly used test setups found in literature: **(A)** Oxford-rig, **(B)** horizontal rig and **(C)** (“modified’’) material testing machines. They differ mainly in force application, knee alignment, and range of motion.

The different test setups clearly indicate that the used hardware and test strategies greatly influence the level and changes of CP values (Figure 10). A non-physiological alignment and embedding of knee specimens can lead to considerably changed and misleading values.

During natural movements, the knee joint offers six degrees of freedom (DOF) (Hirschmann and Muller, 2015). In the case of a limitation of these six DOF, the kinematics of the knee joint is altered, leading also to an altered CP. In addition, depending on the horizontal or vertical alignment of the knee joint during the experiments, the peak CP might also be altered. While the horizontal knee alignment is more likely to eliminate gravitational effects in the flexion-extension plane (Amis et al., 2006), because of its anatomically correct knee joint alignment, the vertical alignment reflects a more physiological load bearing situation. To obtain realistic kinematical parameters that are comparable to the *in vivo* situation, conventional or modified material testing machines, Oxford-rigs, horizontal rigs and wear simulators are used (Supplementary Material). Using conventional material testing machines (Agneskirchner et al., 2007; Marzo and Gurske-Deperio, 2009; Van Thiel et al., 2011; Forkel et al., 2014; Zhang et al., 2015; Brown et al., 2016), the application of an axial load on a vertically aligned knee joint can be readily implemented. Modified material testing machines with customized loading setups are normally able to provide all six DOF during *in vitro* experiments (Becker et al., 2005; Rodner et al., 2006; Schillhammer et al., 2012; Kim et al., 2013; Bryant et al., 2014; Goyal et al., 2014; Lee, 2014). The main components of custom-made vertical knee joint simulators, so-called Oxford-rig simulators (Mcculloch et al., 2016; Steinbruck et al., 2016), are ankle and hip assemblies that also enable an unconstrained movement of the knee joint in all six DOF (Zavatsky, 1997). In Oxford-rigs, a horizontal actuator normally simulates the flexion-extension movements without simulating an according axial or ground reaction force. Another type of the experimental setup is a horizontal rig (Stukenborg-Colsman et al., 2002; Ostermeier et al., 2006; Hofer et al., 2012), where the specimens are mounted with the femur or the tibia fixed horizontally and the patella facing up- or downwards (Stukenborg-Colsman et al., 2002). Movement of the tibia or femur is achieved either passively by an actuator or actively by means of active flexor and extensor muscle simulation, such as quadriceps or hamstring muscle. Additional muscle forces can be also simulated with dead weights. Lastly, CP can be investigated using a knee joint wear simulator (Bedi et al., 2010), which is normally used to examine different knee replacement designs. Such commercial simulators focus on long-term gait simulations, providing six DOF and flexion angles of up to 60°, while the flexion angle, axial force, anterior-posterior shear force or internal-external torque can be varied (Walker et al., 2000; van Houtem et al., 2006) accordingly.

Of course, the kinematical configuration of a test setup plays a crucial role when investigating the peak CP, where it is either possible to simulate dynamic (e.g., gait cycles, drop jump, etc.), static (e.g., fixed knee flexion angle) or so-called quasi-static (e.g., very slow simulation of predefined flexion-extension cycles) loading situations. Several groups used fixed knee flexion angles to simplify the testing apparatus (Marzo and Gurske-Deperio, 2009; Van Thiel et al., 2011; Forkel et al., 2014; Brown et al., 2016). The fixed angles and pre-defined axial loads allow groups to investigate research questions and their effects on knee kinematics without additional expense or new machine investment. Complex setups like Oxford-rigs, by contrast, offer greater dynamics and the simulation of muscle forces and motion patterns (Bryant et al., 2014; Mcculloch et al., 2016; Steinbruck et al., 2016; Schall et al., 2019).

### External Load Application

An adequate load application is essential when attempting to simulate *in vivo* knee joint loading conditions and, therefore, CP values at a realistic pathological or physiological level. Loads can be applied either directly *via* the bony structure (*isolated axial load*) by the loading apparatus, indirectly by *muscle force simulation*, or using a combination of both.

#### Axial Load

One of the most frequently applied loads is the (isolated) axial load, which can be applied either statically or dynamically. During pure axial loading situations, the knee flexion angle needs to be fixed at a previously defined position, otherwise the knee would not be loaded but rather flexed into a different position. Differences in applied isolated axial loads to the knee joints ranged from 0 up to 2,700 N (Lee et al., 2006; Bode et al., 2017; van Egmond et al., 2017).

Instead of isolated axial loads, extension moments or a combination of isolated axial load and external moments can be applied (Stukenborg-Colsman et al., 2002; Ostermeier et al., 2006; Bedi et al., 2010) to simulate gait cycles or isokinetic extension movements (Amadi et al., 2008; Bode et al., 2017). Additionally, a combination of isolated axial loads or external moments and muscle force simulations (Stukenborg-Colsman et al., 2002; Becker et al., 2005; Ostermeier et al., 2006; Hofer et al., 2012; Goyal et al., 2014; Lee, 2014; Mcculloch et al., 2016; Steinbruck et al., 2016), as well as variable muscles force simulations can be used (Bryant et al., 2014) to simulate different knee joint loading situations.

Peak CP measurements during joint weight-bearing is quite challenging (Rodner et al., 2006). In some studies, the applied forces were not able to achieve physiological loading conditions (Rodner et al., 2006; Schillhammer et al., 2012; Kim et al., 2013; Forkel et al., 2014; Mcculloch et al., 2016), resulting in a comparably lower peak CP and thus, being unable to simulate realistic load scenarios that occur during daily activities (Kim et al., 2013). Baratz et al. (1986), Paletta et al. (1997), Ode et al. (2012), and Dienst et al. (2007) measured CP after performing a total meniscectomy using material testing machines with different axial loads but with comparable test setups. Baratz et al. and Paletta et al. applied 1,800 N of axial load, whereas Ode et al. and Dienst et al. used an axial force of 800 and 1,000 N, respectively. Between healthy knee joints and total meniscectomized joints, Baratz et al. and Paletta et al. measured an increase in the peak CP of 100–235%. Ode et al. and Dienst et al. reported a more moderate increase in the peak CP of less than 50% after total meniscectomy. On the basis of only the differences in axial loads, it can be assumed that these differences are mainly attributed to the different axial loading conditions.

To investigate the influence of limb malalignments, Willinger et al. loaded their cadaveric knees axially with 750 N (Willinger et al., 2019) in neutral, varus and valgus alignments. They demonstrated that varus alignment significantly increased the medial peak CP compared to neutral or valgus alignment, with an intact medial meniscus. By contrast, valgus malalignment and a neutral axis resulted in a reduced medial CP. This findings were supported by Agneskirchner et al. (2007), Bruns et al. (1993) and Inaba et al. (1990). Summing up the results of these studies, all authors concluded that during varus malalignment, the medial compartment is loaded significantly more than the lateral compartment. Whereas in a valgus position, the ratios are consequently reverse.

#### Muscle Force Simulation

Muscle force simulation is important to achieve realistic and physiological loading conditions, particularly during dynamic *in vitro* knee joint simulations. The muscle forces stabilize the knee joint (Forkel et al., 2014) and are key for generating a dynamic compressive tibiofemoral force, for example, during knee flexion and extension (Bryant et al., 2014). Differences in muscle force simulation are due to the number of simulated muscles and the magnitude of the applied loads (Stukenborg-Colsman et al., 2002; Becker et al., 2005; Ostermeier et al., 2006; Hofer et al., 2012; Bryant et al., 2014; Goyal et al., 2014; Lee, 2014; Mcculloch et al., 2016; Steinbruck et al., 2016). Muscles can be simulated either *via* pneumatic or hydraulic actuators or simplified *via* dead weights. Simulation *via* actuators allows adjustment of the muscle forces over time or to be related to the knee flexion angle. However, due to the difficulty of a suitable control for the actuators (Maletsky and Hillberry, 2005; Muller et al., 2009) or challenges at the connection between muscle or tendon and the actuator itself, the simulated muscle forces are frequently lower than those encountered *in vivo* (Hofer et al., 2012). Abrupt increases in forces or dynamic gait scenarios frequently lead to tearing or slipping of fasteners or tissue grips. This finally leads to an underrepresentation of *in vivo* contact mechanics (Hofer et al., 2012; Bryant et al., 2014). A lack of variability of the muscle forces with varying knee flexion angle, particularly when using dead weights, and in different knee joint states might also lead to altered, mostly underestimated CP results (Li et al., 2002; Hofer et al., 2012). In principle, the muscle force simulation should be adapted to the *in vivo* muscle activation profile of the specific movement. These datasets can be acquired by inverse dynamic models that are based on kinematic and kinetic measurements in professional gait laboratories (Alimusaj et al., 2009) and even *via* open source database (e.g., OrthoLoad.com). Additionally, the simulation of agonist-antagonist interaction of the respective muscle groups needs to be considered when simulating dynamic recurring movements, for example, gait cycles. Otherwise, the knee joint is loaded insufficiently, leading to an underestimated CP. Ostermeier et al. found an increase in the peak CP of up to 15% of the initial peak CP with quadriceps muscle force and additional co-contraction of the hamstring muscles compared to single quadriceps muscle simulations (Ostermeier et al., 2006). The co-contraction of the simulated muscles thereby pulls the tibial compartment more strongly against the femoral condyle.

## Discussion

The aim of this systematic review was to investigate the influence of menisci on tibiofemoral contact mechanics in human knees and to summarize the influence of various test setups, load applications and muscle force simulations on the determination of the tibiofemoral CP after simulated meniscal injuries and their surgical treatments, including sutures and meniscectomies. This study was also performed to describe the effects of CP-elevating events and thereby explain the increased risk for patients to develop PTOA and how this can be delayed or even prevented.

### Meniscus Pathology

The results of our systematic literature review indicated, that an injured meniscus, for example, meniscal tears, leads to significantly increased CPs within the knee joint. The peak CP values increase up to 235% in total meniscectomized knees compared to their intact meniscal state (Baratz et al., 1986). The analysis of the published peak CPs showed a wide range from 0.1 MPa (van Egmond et al., 2017) to 12.06 MPa (Poh et al., 2012). Surgical treatments like sutures or meniscus replacements are able to restore the CP almost to the initial intact state. Degenerated or pathologically altered menisci lead to increased peak CPs and their repair is necessary to ideally restore the native CP distribution. This is essential because it is widely accepted that meniscal alteration, and thus an increased peak CP, is one crucial factor initiating the early onset of PTOA (Badlani et al., 2013; Thomas et al., 2017). Therefore, one primary treatment goal of a meniscus pathology is to restore the CP close to the intact state and ideally rebalance the load distribution between the lateral and medial compartments.

In summary, post-traumatic changes of the meniscus significantly increase the CP in the knee. Tears and partial or total meniscectomy treatments lead to increased CP in the knee joint, whereas repairing techniques, like meniscus sutures, are able to restore the CP almost similar to the intact meniscal state. The publications evaluated in this review widely coincide in their outcomes, particularly after a meniscal injury when the peak CP tendentially increases, whereas it can be restored by surgical treatment. Moreover, the published CP values showed clear trends after an injury or its treatment.

However, it is also clear from the present study that the absolute values of the reported CPs cannot be compared. The different approaches of the research groups make it difficult to determine the “truth” of the CP. Therefore, it more practical to compare the relative values, while real values (in MPa) are more likely to lead to non-comparable values, particularly because of the large number of different test setups and simulations. What has become clear, however, is the fact that untreated meniscal injuries inevitably lead to a significant increase in CP and in the long term are highly prone to progress to PTOA.

### Testing Conditions

As indicated above, large differences were found in the application of loads and the alignment of the knee joint specimens in different test setups. It could be demonstrated that greater applied axial loads lead to a higher peak CP and muscle force simulation resulted in a more physiological knee joint loading condition with an accordingly higher peak CP. Muscle force simulation leads to a more realistic environment of the knee joint in *in vitro* testing. On the one hand, the knee is loaded multidimensionally, and on the other hand, the knee is held and guided more stably in the test environment. This results in a more physiological outcome compared to pure axial loads. The test setups and their respective load simulations in *in vitro* peak CP measurements indicated a remarkable axial loading range from 0 to 2,700 N. It appears clear that greater axial loads and applied muscle force simulations lead to higher CP, which makes it difficult for an objective comparison of the so gathered CP values. Values obtained through the different research approaches cannot be compared with each other. This can be seen, for example, in the comparison of the two studies with similar test setups by Agneskirchner et al. (isolated axial load 1,000 N) and Inaba et al. (isolated axial load 2,700 N), with peak CPs of 1.72 MPa (Agneskirchner et al., 2007) vs. 4.01 MPa (Inaba et al., 1990) shown in the intact knee state with normal alignment. Furthermore, Huang et al. demonstrated that pressure differences cannot be scaled up from lower load scenarios to higher ones (Huang et al., 2003). Rather, the trend of the peak CP outcome in each study needs to be evaluated, as well as its general research question. This can be used, for example, to determine the success of a treatment strategy, but not to investigate questions in which the magnitude of the peak CP is important. This could be the case, for example, when investigating the stability of load-critical replacements or implants.

In addition, lower limb varus malalignment leads to an increased medial CP, which is further considered as a promoter of premature OA (Inaba et al., 1990; Bruns et al., 1993; Agneskirchner et al., 2007; Willinger et al., 2020). The limb alignment must be considered in each test, because this elementary parameter can have a major effect on the result by increasing or decreasing CPs unintentionally. Therefore, care must be taken to ensure that the clamping is performed in a native manner without placing the knee unintentionally in a malalignment and thus influencing the peak CP.

Therefore, we propose recommendations for future *in vitro* testing of knee joint contact mechanics. This was done to obtain the most realistic *in vivo* data possible from testing in addition to the already gathered significant trends. This is also intended to make biomechanical studies more comparable, objective and consistent in approach and findings.

### Recommendations for Future Biomechanical CP Studies

A biomechanically based recommendation regarding the experimental procedures for *in vitro* investigations on CP of the knee joint focusing on meniscus modifications is presented in Table 2, including the requirements for the test setup, static or dynamic testing methods, magnitude and direction of the applied loads and muscle force simulation. Here, the physiological vertical knee alignment is the key factor. The test setup should be extended by the knee-stabilizing quadriceps and hamstrings muscles and an axial load adapted to the specimen (*in vivo* weight simulation). For the kinematic examination, normal daily exercises with flexion and extension (e.g., gait cycle) should be selected (Huang et al., 2003).

**TABLE 2:** Recommendations for the determination of contact mechanics regarding the meniscus and meniscal injuries.

Test setup	Motion	Load	Muscle simulation
Modified material testing machine	Flexion-extension cycle with varying flexion rate	Axial load (body weight)	Quadriceps
Oxford-rig		External moments	Hamstring
Horizontal rig			

Due to the time-dependent responses of meniscal tissue and its dynamic environment within the knee joint, motion of the knee joint may be better suited to investigate CP with meniscal tears or after meniscus resection (Goyal et al., 2014). The range of motion and the velocity of the motion should be as close as possible to the physiological movement of a human knee joint seen during activities of daily life. Ideally, the axial load should be either combined with a sufficient muscle force simulation or alternatively tibiofemoral contact forces should result from such an appropriate muscle force simulation. The major knee spanning muscles - quadriceps and the hamstrings - are particularly important for the stabilization of the knee joint and should be simulated. The preferred test setups are horizontal rigs, Oxford-rigs and modified material testing machines, in particular Oxford-rigs and material testing machines, because they may have advantages due to their upright knee joint position. The axial load should be related to the biometric data of the donor of the knee joint specimen to allow reasonable *in vivo*-like weight bearing simulation. Static knee joint loading should ideally be avoided, because the highly anisotropic and inhomogeneous viscoelastic menisci show a time- and localization-depending behavior. The latter is mainly determined by the interaction of the menisci with their surrounding soft tissues within the knee joint. Therefore, we recommend to rather simulate dynamic loading situations with varying knee joint flexion angles as seen during activities of daily life, like e.g. normal gait or stair climbing. However, dynamic testing is not possible in every research institution. If dynamic testing, as suggested by us, is not possible, static tests with different axial load profiles and different flexion angles should be performed. Additionally, the axial load and limb alignment should be adapted to the donor as a minimum requirement.

Considering this, it may lead to greater transparency and, therefore, an increased knowledge in the understanding of knee joint contact mechanics. When consistent test setups and test procedures are introduced and implemented by research institutions, different fields can thereby be investigated and a wide-ranging field of interest with a high level of knowledge exchange could be obtained.

At the very least, the description of the experimental procedure should be presented in a reproducible and thus fully transparent way. The description of the test setup, test procedure, load application, and muscle force simulation should be made very precisely to be able to reproduce tests and their results. Such a description should at least incorporate the following terms: Description of test setup and included modifications, DOF at all bearings, fixation of femur, and tibia, actuators, sensors, and the used control mode; for static testing all tested flexion angles should be mentioned; for motion cycles, both, the range and velocity of the motion should be indicated; direction and magnitude of the applied loads must be known. Information about muscle simulation should include the simulated muscle groups, simulation type with weights or actuators (control mode), magnitude and direction of the simulated muscles and a description of the change of magnitude over time or flexion angle.

## Data Availability

The original contributions presented in the study are included in the article/Supplementary Material, further inquiries can be directed to the corresponding author.
