# Supplementary material for: Influence of Menisci on Tibiofemoral Contact Mechanics in Human Knees: A Systematic Review
Source: Front Bioeng Biotechnol. 2021 Dec 3;9:765596. doi: 10.3389/fbioe.2021.765596 (PMC8681859; doi:10.3389/fbioe.2021.765596)
Supplement: Supplementary file 1 [file DataSheet2.PDF]

Data from publications, approximated from graphs

Article	Study	Flexion	Load in N / Torque in Nm	Stage (medial/lateral)	Peak contact pressure (CP) in MPa
Allaire et al. (2008)	meniscal root tear (medial)	0°	1000N	intact	5,3
		0°	1000N	PMMR repair	4,95
		0°	1000N	PMMR tear	5,15
		0°	1000N	total menisectomy	5,1
		30°	1000N	intact	4,5
		30°	1000N	PMMR repair	4,75
		30°	1000N	PMMR tear	5,95
		30°	1000N	total menisectomy	6,6
		60°	1000N	intact	5,5
		60°	1000N	PMMR repair	5,7
		60°	1000N	PMMR tear	7,6
		60°	1000N	total menisectomy	7,3

Baratz et al. (1986)	tear, repair, menisectomy	0°	1800N	intact (m)	1,4
		0°	1800N	partial menisectomy (m)	2,2
		0°	1800N	total menisectomy (m)	4,6
		0°	1800N	intact (m)	1,4
		0°	1800N	peri tear (m)	1,7
		0°	1800N	closed repair (m)	1,5
		0°	1800N	open repair (m)	1,4
		0°	1800N	segmental menisectomy (m)	3
		0°	1800N	total menisectomy (m)	4,1
		0°	1800N	intact (l)	2,5
		0°	1800N	peri tear (l)	2,6
		0°	1800N	closed repair (l)	2,4
		0°	1800N	open repair (l)	2,5
		0°	1800N	segmental menisectomy (l)	4,8
		0°	1800N	total menisectomy (l)	5,2

		30°	1800N	intact	1,3
		30°	1800N	partial menisectomy	1,8
		30°	1800N	total menisectomy	3,8
Beamer et al. (2017)	meniscal tear (medial)	0°	1310-2290N	intact	3,6
		0°	1310-2290N	tear	6,3
		0°	1310-2290N	repair	3,9
		0°	1310-2290N	partial resection	5,9
		0°	1310-2290N	full resection	8,1
Chen Z. et al. (2020)	meniscal repair	0°	1000N	Native (m)	4
		30°	1000N	Native(m)	4,1
		60°	1000N	Native(m)	5,4
		90°	1000N	Native(m)	6
		0°	1000N	vertical longitudinal tear(m)	5,6
		30°	1000N	vertical longitudinal tear(m)	6,8
		60°	1000N	vertical longitudinal tear(m)	6,2
		90°	1000N	vertical longitudinal tear(m)	7,8
		0°	1000N	Repaired mattress suture(m)	4,3
		30°	1000N	Repaired mattress suture(m)	4,2
		60°	1000N	Repaired mattress suture(m)	5,6
		90°	1000N	Repaired mattress suture(m)	8
Huang et al. (2003)	lateral meniscal autograft	0°	400N	intact	3
		0°	400N	autograft	3,1
		0°	400N	menisectomy	4,2
		0°	1200N	intact	5,1
		0°	1200N	autograft	8,3

		0°	1200N	menisectomy	8,4
		30°	400N	intact	3
		30°	400N	autograft	3
		30°	400N	menisectomy	3,2
		30°	1200N	intact	5,8
		30°	1200N	autograft	7,1
		30°	1200N	menisectomy	8,2

Marchetti et al. (2017)	tear repair (all inside/inside out)	0°	1000N	intact	2,2
		0°	1000N	MCLR	2,2
		0°	1000N	Meniscus Cut	2,8
		0°	1000N	in-out Repair	2,2
		0°	1000N	All.in Repair	2,5
		30°	1000N	intact	1,9
		30°	1000N	MCLR	2
		30°	1000N	Meniscus Cut	3,6
		30°	1000N	in-out Repair	2,3
		30°	1000N	All.in Repair	2,3
		60°	1000N	intact	2,3
		60°	1000N	MCLR	2,3
		60°	1000N	Meniscus Cut	4,9
		60°	1000N	in-out Repair	2,9
		60°	1000N	All.in Repair	2,8

McDermott et al. (2008)	meniscal allograft (lateral)	0°	700N	Intact	5,3
		0°	700N	Menisectomy	7,6
		0°	700N	Bone plug	6
		0°	700N	Sutures only	6,3

Muriuki et al. (2011)	meniscal split (radial)	0°	1000N	Intact (m)	5,6
	"	0°	1000N	Tom Meniscus(m)	5,2
	"	0°	1000N	Repair(m)	4,9
	"	0°	1000N	Total menisectomy(m)	6,8
	"	30°	1000N	Intact(m)	5,6
	"	30°	1000N	Tom Meniscus(m)	5,6
	"	30°	1000N	Repair(m)	5,3
	"	30°	1000N	Total menisectomy(m)	8,5
	"	60°	1000N	Intact(m)	6,3
	"	60°	1000N	Tom Meniscus(m)	6,2
	"	60°	1000N	Repair(m)	6,9
	"	60°	1000N	Total menisectomy(m)	9,1
	"	0°	1000N	Intact(l)	5,6
	"	0°	1000N	Tom Meniscus(l)	5,9
	"	0°	1000N	Repair(l)	5,3
	"	0°	1000N	Total menisectomy(l)	6,2
	"	30°	1000N	Intact(l)	5,9
	"	30°	1000N	Tom Meniscus(l)	5,2
	"	30°	1000N	Repair(l)	4,5
	"	30°	1000N	Total menisectomy(l)	4,6
	"	60°	1000N	Intact(l)	5,5
	"	60°	1000N	Tom Meniscus(l)	5,7
	"	60°	1000N	Repair(l)	5,6
	"	60°	1000N	Total menisectomy(l)	4,9
	vertical cleavage	0°	1000N	Intact (m)	4,1
	"	0°	1000N	Tom Meniscus(m)	5,6
	"	0°	1000N	Repair(m)	4,3
	"	0°	1000N	Total menisectomy(m)	5,9
	"	30°	1000N	Intact(m)	4,8
	"	30°	1000N	Tom Meniscus(m)	5,5
	"	30°	1000N	Repair(m)	5
	"	30°	1000N	Total menisectomy(m)	7
	"	60°	1000N	Intact(m)	5

	"	60°	1000N	Tom Meniscus(m)	6,1
	"	60°	1000N	Repair(m)	5,2
	"	60°	1000N	Total menisectomy(m)	6,5
	"	0°	1000N	Intact(l)	4,2
	"	0°	1000N	Tom Meniscus(l)	5,1
	"	0°	1000N	Repair(l)	4,5
	"	0°	1000N	Total menisectomy(l)	4,9
	"	30°	1000N	Intact(l)	4,2
	"	30°	1000N	Tom Meniscus(l)	5,4
	"	30°	1000N	Repair(l)	5
	"	30°	1000N	Total menisectomy(l)	5,5
	"	60°	1000N	Intact(l)	5,3
	"	60°	1000N	Tom Meniscus(l)	5,4
	"	60°	1000N	Repair(l)	5,45
	"	60°	1000N	Total menisectomy(l)	5,9

Paci et al. (2009)	intact vs. AIML cut (m)	0°	1000N	intact	2,4
		30°	1000N	intact	3,4
		60°	1000N	intact	4,5
		0°	1000N	AIML cut	2,8
		30°	1000N	AIML cut	4,1
		60°	1000N	AIML cut	5

Prince et al. (2014)	intact, tear, repair, menisctomy	0°	1000N	intact (m)	2,9
		0°	1000N	AHLM tear (m)	2,7
		0°	1000N	repaired (m)	2,4
		0°	1000N	menisectomy (m)	3,4
		0°	1000N	intact (l)	2,6
		0°	1000N	AHLM tear (l)	2,6
		0°	1000N	repaired (l)	2,5

		0°	1000N	menisectomy (l)	3,6
		30°	1000N	intact (l)	1,9
		30°	1000N	AHLM tear (l)	2
		30°	1000N	repaired (l)	1,9
		30°	1000N	menisectomy (l)	3,4

Seitz et al. (2012)	partial menisectomy lateral/medial	0°	500N	intact	1,8
		0°	500N	20%	2
		0°	500N	50%	1,5
		0°	500N	100%	1,9
		30°	500N	intact	1,5
		30°	500N	20%	1,2
		30°	500N	50%	1,5
		30°	500N	100%	1,75
		60°	500N	intact	1,8
		60°	500N	20%	2
		60°	500N	50%	2,1
		60°	500N	100%	2,5
		0°	500N	intact (l)	1,85
		0°	500N	20%(l)	1,9
		0°	500N	50%(l)	2
		0°	500N	100%(l)	2,6
		30°	500N	intact(l)	1,9
		30°	500N	20%(l)	2
		30°	500N	50%(l)	2,1
		30°	500N	100%(l)	3
		60°	500N	intact(l)	2
		60°	500N	20%(l)	2,25
		60°	500N	50%(l)	2,3
		60°	500N	100%(l)	2,9

Stein et al. (2019)	intact, partial medial minesectomy, partial meniscal replacement	0°	2000N	intact (m)	2
		0°	2000N	partial menisectomy (m)	4
		0°	2000N	partial meniscal replacement	1,9
		30°	2000N	intact (m)	3,8
		30°	2000N	partial menisectomy (m)	6,5
		30°	2000N	partial meniscal replacement	3,7
		60°	2000N	intact (m)	3,6
		60°	2000N	partial menisectomy (m)	8,8
		60°	2000N	partial meniscal replacement	4,9
