# Supplementary material for: Influence of Menisci on Tibiofemoral Contact Mechanics in Human Knees: A Systematic Review
Source: Front Bioeng Biotechnol. 2021 Dec 3;9:765596. doi: 10.3389/fbioe.2021.765596 (PMC8681859; doi:10.3389/fbioe.2021.765596)
Supplement: Supplementary file 2 [file DataSheet3.pdf]

Data from publications, exact values from the text

Article	Study	Flexion	Load in N / Torque in Nm	Stage (medial/lateral)	Peak contact pressure (CP) in MPa
Agneskirchner et al. (2007)	intact, HTO - varus/valgus	0°	1000N	Neutral (m)	1,72
		0°	1000N	Neutral (l)	2,92
		0°	1000N	Varus (m)	1,47
		0°	1000N	Varus (l)	2,04
		0°	1000N	Valgus (m)	1,43
		0°	1000N	Valgus (l)	2,97
		0°	1000N	HTO (9 mm) (m)	3,78
		0°	1000N	HTO (9 mm) (l)	1,35
		0°	1000N	HTO (9 mm) + MCL release (m)	1,42
		0°	1000N	HTO (9 mm) + MCL release (l)	2,23
Amadi et al. (2008)	intact, meniscofemoral ligaments	0°	700N	4 dof Intact MFL (l)	4,34
		0°	700N	4 dof Intact MFL absent (l)	4,78
		0°	700N (5 Nm ir)	3 dof Intact MFL (l)	4,50
		0°	700N (5 Nm ir)	3 dof Intact MFL absent (l)	4,91
Becker et al. (2005)	intact, meniscal Repair	0°	1400N	Intact (m)	3,12
		0°	1400N	Intact (l)	2,3
		0°	1400N	Fastener Implant (m)	2,86
		0°	1400N	Fastener Implant (l)	3,58
Bode et al. (2017)	HTO	0°	31 Nm	nativ (m)	1,19
		0°	31 Nm	absorber (m)	0,99
		0°	31 Nm	HTO 5 (m)	0,92

		0°	31 Nm	HTO 10 (m)	0,60
		30°	31 Nm	nativ (m)	0,99
		30°	31 Nm	absorber (m)	0,74
		30°	31 Nm	HTO 5 (m)	0,58
		30°	31 Nm	HTO 10 (m)	0,37
		60°	31 Nm	nativ (m)	0,98
		60°	31 Nm	absorber (m)	0,75
		60°	31 Nm	HTO 5 (m)	0,56
		60°	31 Nm	HTO 10 (m)	0,30
		90°	31 Nm	nativ (m)	0,98
		90°	31 Nm	absorber (m)	1,18
		90°	31 Nm	HTO 5 (m)	0,83
		90°	31 Nm	HTO 10 (m)	1,02

Brown et al. (2016)	meniscal tear/meniscectomy	0°	1800N	Native (m)	2,1
		0°	1800N	Native (l)	4,51
		0°	1800N	Tear (m)	2,03
		0°	1800N	Tear (l)	4,5
		0°	1800N	Inferior leaflet (m)	2,03
		0°	1800N	Inferior leaflet (l)	4,5
		0°	1800N	Both leaflet (m)	2,8
		0°	1800N	Both leaflet (l)	4,86

Bruns et al. (1993)	meniscal Repair/meniscectomy	0°	500N	neutral intact (m)	3,68
		0°	500N	neutral intact (l)	2,9
		0°	500N	10° varus intact (m)	5,33
		0°	500N	10° varus intact (l)	2,04
		0°	500N	10° valgus intact (m)	2,77

		0°	500N	10° valgus intact (l)	4,18

Bryant et al. (2014)	TKA - neutral/5° valgus	0°	---	Neutral TKA (l)	6
		0°	---	Valgus TKA (l)	7,7
		30°	---	Neutral (l)	5,7
		30°	---	5° Valgus (l)	8,4
		60°	---	Neutral (l)	4,7
		60°	---	5° Valgus (l)	8,1

Dugas et al. (2015)	intact, meniscocapsular mepair	0°	1500N	intact (m)	2,59
		0°	1500N	meniscocapsular separation (m)	3,03
		0°	1500N	repair (m)	2,84

Forkel et al. (2014)	intact, meniscal Tear	0°	100N	Intact (l)	0,44
		0°	100N	Root Tear (l)	0,54
		0°	100N	Repair (l)	0,51

Geeslin et al. (2016)	intact, meniscal Repair/meniscectomy	0°	1000N	intact (l)	2,77
		30°	1000N	intact (l)	2,15
		60°	1000N	intact (l)	2,38

Goyal et al. (2014)	intact, meniscal tear/meniscectomy	0°	350N	Intact (l)	7,6
		0°	350N	Vertical Tear (l)	8,3
		0°	350N	Partial Meniscectomy (l)	5,4

		0°	350N	Total Meniscectomy (l)	5,6
		30°	350N	Intact (l)	7,9
		30°	350N	Vertical Tear (l)	8,1
		30°	350N	Partial Meniscectomy (l)	8,1
		30°	350N	Total Meniscectomy (l)	8,4
		60°	350N	Intact (l)	7,7
		60°	350N	Vertical Tear (l)	7,8
		60°	350N	Partial Meniscectomy (l)	8,7
		60°	350N	Total Meniscectomy (l)	8,4

Inaba et al. (1990)	intact,meniscectomy	0°	2700N	Neutral 0° / intact (m)	4,01
		0°	2700N	Neutral 0° / intact (l)	4,12
		0°	2700N	varus 5° / intact (m)	7,32
		0°	2700N	varus 5° / intact (l)	0,41
		0°	2700N	valgus 5° / intact (m)	0
		0°	2700N	valgus 5° / intact (l)	7,83
		0°	2700N	Neutral 0° / menisectomy (m)	6,74
		0°	2700N	Neutral 0° / menisectomy (l)	7,36
		0°	2700N	varus 5° / menisectomy (m)	8,12
		0°	2700N	varus 5° / menisectomy (l)	0
		0°	2700N	valgus 5° / menisectomy (m)	0
		0°	2700N	valgus 5° / menisectomy (l)	9,23

LaPrade et al. (2015)	meniscal repair (all medial)	0°	1000N	intact	2,86
		0°	1000N	root tear	3,75
		0°	1000N	anatomic repair	3,63
		0°	1000N	nonanatomic repair	3,98
		0°	1000N	sectioned ACL	4,20
		30°	1000N	intact	2,56

		30°	1000N	root tear	4,20
		30°	1000N	anatomic repair	3,40
		30°	1000N	nonanatomic repair	4,38
		30°	1000N	sectioned ACL	7,58
		60°	1000N	intact	3,61
		60°	1000N	root tear	5,49
		60°	1000N	anatomic repair	4,51
		60°	1000N	nonanatomic repair	5,60
		60°	1000N	sectioned ACL	7,26
		90°	1000N	intact	3,60
		90°	1000N	root tear	5,76
		90°	1000N	anatomic repair	4,43
		90°	1000N	nonanatomic repair	6,12
		90°	1000N	sectioned ACL	7,56

Lee et al. (2006)	intact, meniscectomy (all medial)	0°	1800N	intact	4,47
		0°	1800N	50 % meniscectomy	5,94
		0°	1800N	75 % meniscectomy	7,17
		0°	1800N	segmental	8,66
		0°	1800N	total	9,27
		30°	1800N	intact	3,10
		30°	1800N	50 % meniscectomy	4,51
		30°	1800N	75 % meniscectomy	5,65
		30°	1800N	segmental	6,90
		30°	1800N	total	7,49
		60°	1800N	intact	4,35
		60°	1800N	50 % meniscectomy	6,47
		60°	1800N	75 % meniscectomy	7,99
		60°	1800N	segmental	9,39
		60°	1800N	total	10,03

Marzo et al. (2009)	intact, meniscal horn tear/repair	0°	1800N	Intact (m)	3,84
		0°	1800N	Intact (l)	5,08
		0°	1800N	Posterior Horn Tear (m)	5,08
		0°	1800N	Posterior Horn Tear (l)	5,23
		0°	1800N	Repair (m)	3,55
		0°	1800N	Repair (l)	5,33

Perez-Blanca et al. (2016)	intact	0°	1000N	intact (l)	3,63
		0°	1000N	intact (m)	3,94
		30°	1000N	intact (l)	3,12
		30°	1000N	intact (m)	4,84
		60°	1000N	intact (l)	3,29
		60°	1000N	intact (m)	5,32
		90°	1000N	intact (l)	3,68
		90°	1000N	intact (m)	5

Poh et al. (2012)	intact, anterior intermeniscal ligament	0°	1800N	intact presectioning (m)	11,7
		0°	1800N	intact presectioning (l)	6,89
		0°	1800N	postsectioning (m)	12,06
		0°	1800N	postsectioning (l)	7,11

Rodner et al. (2006)	intact, HTO	0°	500N	HTO (m)	2,8
		0°	500N	HTO (l)	3,3
		30°	500N	HTO (m)	2,7
		30°	500N	HTO (l)	2,5

Seitz et al. (2019)	HTO and medial collateral ligament	0°	1000N	neutral / intact MCL (l)	2,15
		0°	1000N	neutral / released MCL (l)	1,43
		0°	1000N	5° HTO / intact MCL (l)	1,91
		0°	1000N	5° HTO / released MCL (l)	1,61
		0°	1000N	10° HTO / intact MCL (l)	1,18
		0°	1000N	10° HTO / released MCL (l)	1,83
		0°	1000N	neutral / intact MCL (m)	1,67
		0°	1000N	neutral / released MCL (m)	2,16
		0°	1000N	5° HTO / intact MCL (m)	1,83
		0°	1000N	5° HTO / released MCL (m)	1,97
		0°	1000N	10° HTO / intact MCL (m)	2,5
		0°	1000N	10° HTO / released MCL (m)	2,1
		30°	1000N	neutral / intact MCL (l)	1,69
		30°	1000N	neutral / released MCL (l)	1,31
		30°	1000N	5° HTO / intact MCL (l)	1,38
		30°	1000N	5° HTO / released MCL (l)	1,34
		30°	1000N	10° HTO / intact MCL (l)	1,23
		30°	1000N	10° HTO / released MCL (l)	1,27
		30°	1000N	neutral / intact MCL (m)	2,69
		30°	1000N	neutral / released MCL (m)	2,03
		30°	1000N	5° HTO / intact MCL (m)	2,32
		30°	1000N	5° HTO / released MCL (m)	2,12
		30°	1000N	10° HTO / intact MCL (m)	2,68
		30°	1000N	10° HTO / released MCL (m)	2,22

van Egmond et al. (2017)	HTO / MCL	0°	n.a	intakt (m)	0,1
		0°	n.a	intakt (l)	0,11
		0°	n.a	Osteotomy 10° (m)	0,38

		0°	n.a	Osteotomy 10° (l)	0,03
		0°	n.a	MCL release 10°(m)	0,11
		0°	n.a	MCL release 10°(l)	0,13

Van Thiel et al. (2011)	intact, meniscectomy, transplant	0°	800N	Neutral Intact (m)	1,91
		0°	800N	Neutral Meniscectomy (m)	2,71
		0°	800N	Neutral Transplant (m)	2,12
		0°	800N	6° Varus Intact (m)	2,73
		0°	800N	6° Varus Meniscectomy (m)	3,41
		0°	800N	6° Varus Transplant (m)	3,05
		0°	800N	6° Valgus Intact (m)	0,85
		0°	800N	6° Valgus Meniscectomy (m)	1,31
		0°	800N	6° Valgus Transplant (m)	0,9

Verma et al. (2008)	intact, meniscus Repair / meniscectomy (medial)	0°	1000N	intact	5,06
		0°	1000N	menisectomy	8,78
		0°	1000N	bone plug	7,83
		0°	1000N	bone trough	4,95

Willinger et al. (2020)	intact, meniscus / meniscectomy (medial)	0° / 70% valgus	750N	intact	1,5
		0° / 70% valgus	750N	50% resection	1,74
		0° / 70% valgus	750N	75% resection	1,94
		0° / 70% valgus	750N	total menisectomy	2,18
		0° / 60 % valgus	750N	intact	1,66
		0° / 60 % valgus	750N	50% resection	2,12
		0° / 60 % valgus	750N	75% resection	2,44

		0° / 60 % valgus	750N	total menisectomy	2,67
		0° / 50% neutral	750N	intact	2,08
		0° / 50% neutral	750N	50% resection	2,59
		0° / 50% neutral	750N	75% resection	2,63
		0° / 50% neutral	750N	total menisectomy	2,87
		0° / 40% varus	750N	intact	2,66
		0° / 40% varus	750N	50% resection	3,1
		0° / 40% varus	750N	75% resection	3,43
		0° / 40% varus	750N	total menisectomy	3,62
		0° / 30% varus	750N	intact	3,39
		0° / 30% varus	750N	50% resection	3,96
		0° / 30% varus	750N	75% resection	4,33
		0° / 30% varus	750N	total menisectomy	4,58

Zhang et al (2015)	intact, meniscus / meniscectomy (medial)	0°	1000	NovoStick repair (m)	0,96
		0°	1000	Inside-out reapir (m)	0,96
		0°	1000	Partial Meniscectomy (m)	1,5
		30°	1000	NovoStick repair (m)	0,83
		30°	1000	Inside-out reapir (m)	0,86
		30°	1000	Partial Meniscectomy (m)	1,57
