# Supplementary material for: Influence of Menisci on Tibiofemoral Contact Mechanics in Human Knees: A Systematic Review
Source: Front Bioeng Biotechnol. 2021 Dec 3;9:765596. doi: 10.3389/fbioe.2021.765596 (PMC8681859; doi:10.3389/fbioe.2021.765596)
Supplement: Supplementary file 3 [file DataSheet1.pdf]

Supplementary material

List of authors, included in the systematic search.

**Author**	**Study** (meniscal states, ligaments, interventions)	**Test setup**	**Knee flexion angle**	**Motion**	**Load**	**Muscle simulation**	**Pressure measurement**
1. *Agneskirchen et al. (2007)*	***H**igh**T**ibial**O**steotomy (HTO), valgus alignment*	*material testing machine*	*0°*	*N.A.*	*1000 N (axial)*	*N.A.*	*Tekscan*
2. *Ahmed et al. (1983)*	*intact, meniscectomy*	*desktop machine*	*0°, 30°, 60°, 90°*	*N.A.*	*2668 N (axial)*	*N.A.*	*Transducer*
3. *Alhalki et al. (2000)*	*intact, meniscectomy, autograph, allograph*	*horizontal rig*	*0°, 15°, 30°, 45°*	*N.A.*	*1000 N (axial)*	*N.A.*	*Fuji Film*
4. *Allaire et al. (2008)*	*intact, root tear, repair, meniscectomy*	*modified material testing machine*	*0°, 30°, 60°, 90°*	*N.A.*	*1000 N (axial)*	*N.A.*	*Fuji film*
5. *Amadi et al. (2008)*	*meniscofemoral ligaments*	*material testing machine*	*0°*	*N.A.*	*700 N (4 DOF) + 5 Nm int. moment (3 DOF test)*	*N.A.*	*Fuji film*
6. *Andrish et al. (2001)*	*intact*	*modified material testing machine*	*N.A.*	*15° to 75°*	*N.A.*	*Quadriceps ,VL (45 N), VM (45 N), Hamstring (45 N)*	*Force Sensing Technology*
7. *Arno et al. (2015)*	*intact, posterior horn horizontal cleavage lesion*	*desktop machine*	*N.A.*	*−5° to 135°*	*500 N (axial), 100 N P shear, 2.5 Nm torque*	*Quadriceps*	*Tekscan*
8. *Baratz et al. (1986)*	*tear, repair, partial meniscectomy,*	*material testing machine*	*0°, 30°*	*N.A.*	*1800 N (axial)*	*N.A.*	*Fuji Film*
9. *Beamer et al. (2017)*	*intact, horizontal cleavage tear, repair, partial (subtotal) meniscectomy*	*modified material testing machine*	*0°, 10°, 20°*	*N.A.*	*2437 N (axial)*	*N.A.*	*Tekscan*
10. *Becher et al. (2011)*	*intact, with articular resurfacing device*	*Oxford Rig*	*N.A.*	*5° to 45°*	*700 N (GRF)*	*Quadriceps*	*Tekscan*

11. Becker et al. (2005)	*repair of bucket-handle tear*	*modified material testing machine*	*N.A.*	*10° to 90°*	*1400 N (axial)*	*Quadriceps (2000 N)*	*Tekscan*
12. Bedi et al. (2012)	*intact, radial tear, repair, partial meniscectomy*	*wear simulator*	*N.A.*	*gait cycle*	*2100 N (axial), A-P force, rotation torque*	*N.A.*	*Tekscan*
13. Beidokhti et al. (2017)	*intact*	*horizontal rig*	*0°, 30°, 60°, 90°*	*N.A.*	*5.2 Nm torque, 12 Nm, 106 N (axial), 100 N A force*	*RF (20 N), VM (10 N), VL/VI (10N)*	*Tekscan*
14. Bode et al. (2017)	*CP after HTO and absorbing device*	*biomechanical simulator*	*N.A.*	*120° to 0°*	*31 Nm, physiological loading*	*Quadriceps*<br><br>*Hamstring*	*Tekscan*
15. Bretin et al. (2011)	*neutral position, malrotation femur*	*experimental setup with entire lower limb*	*full ext. leg, malrotation 5°,10°,15°. 20°, 25°*	*N.A.*	*half-bodyweight force due to one leg*	*N.A.*	*Tekscan*
16. Brial et al. (2019)	*intact, bone plug meniscal allograft, fixation techniques, meniscectomy*	*multidirectional dynamic simulator*	*N.A.*	*gait cycle*	*physiological loads*	*N.A.*	*Tekscan*
17. Brown et al. (2016)	*horizontal tear, meniscectomy*	*material testing machine*	*0°*	*N.A.*	*1800 N (axial)*	*N.A.*	*Tekscan*
18. Bruns et al. (1993)	*meniscal repair, meniscectomy*	*experimental apparatus*	*0°*	*N.A.*	*500 N (axial)*	*N.A.*	*Fuji Prescale Film*
19. Bryant et al. (2014)	*TKA; normal and valgus*	*experimental apparatus*	*0°. 30°, 60°*	*N.A*	*450 N (muscle load)*	*Quadriceps*	*Fuji Prescale Film*
20. Chen et al. (2016)	*intact, tear, ACL rupture*	*wear simulator*	*N.A.*	*gait cycle*	*2100 N (axial), A-P force, rotation torque*	*N.A.*	*Tekscan*
21. Chen et al. (2020)	*intact, mattress suture repair*	*material testing machine*	*0°, 30°, 60°, 90°*	*N.A.*	*1000 N*<br><br>*5 N-int/ex rotation, 134 N-anterior tibial translation*	*N.A*	*Fuji film*
22. Chen M. et al. (1996)	*meniscectomy, transplantation (different horn fixations)*	*desktop machine*	*0°*	*N.A.*	*310 N (axial)*	*N.A.*	*Inteque Resources Corp.*

This is a provisional file, not the final typeset article

23. Dienst et al. (2007)	*intact, meniscectomy, allografts*	*material testing machine*	*0°, 30°*	*N.A.*	*1000 N (axial)*	*N.A.*	*Fuji film*
24. Du et al. (2017)	*osteochondral allograft (OCA)*	*test setup*	*N.A.*	*N.A*	*Extension moment<br><br>0 Nm, 4Nm, 6 Nm, 8 Nm*	*N.A.*	*Load cell*
25. Dugas et al. (2015)	*meniscocapsular separation, repair*	*modified material testing machine*	*0°*	*N.A.*	*1500 N (axial)*	*N.A.*	*Tekscan*
26. Flemming et al. (2008)	*intact, ACL-reconstruction*	*horizontal fixation test setup*	*0°*	*N.A*	*N.A.<br><br>Passive motion*	*N.A.*	*Tekscan*
27. Forkel et al. (2014)	*lateral posterior root release, transection MFL, refixation posterior root (2 different techniques)*	*material testing machine*	*0°*	*N.A.*	*100 N (axial)*	*N.A.*	*Novel approach*
28. Fukubayashi et al. (1980)	*intact, meniscectomy*	*modified material testing machine*	*0°*	*N.A.*	*1000 N (axial)*	*N.A.*	*Fuji film*
29. Geeslin et al. (2016)	*lateral posterior root avulsion, deficient MFLs, ACL tear, ACL reconstruction, root repair*	*material testing machine*	*0°, 30°, 45°, 60°, 90°*	*N.A.*	*1000 N (axial)*	*N.A.*	*Tekscan*
30. Gilbert et al. (2014)	*intact*	*wear simulator*	*N.A.*	*gait cycle, stair climbin g*	*2100 N (axial), A-P force, rotation torque*	*N.A.*	*Tekscan*
31. Goss et al. (1997)	*anterior cruciate ligament (ACL)*	*experimental apparatus*	*0°, 30°,60°,<br><br>90°, 120°*	*N.A.*	*0–200 N*	*N.A.*	*N.A.*
32. Goyal et al. (2014)	*short (extended) vertical tear, partial (subtotal) meniscectomy*	*modified material testing machine*	*0°, 30°, 60°*	*N.A.*	*350 N (axial)*	*Quadriceps (311 N),Hamstri ng (156 N)*	*Fuji film*
33. Guettler et al. (2007)	*normal, 3°, 6°, 9° varus direction, osteochondral defects*	*modified material testing machine*	*30°*	*N.A.*	*687 N (axial)*	*N.A.*	*Tekscan*
34. Hofer et al. (2012)	*kneeling on TKA*	*custom knee<br><br>testing system*	*90°, 105°, 120°, 135°*	*N.A.*	*339 N /<br><br>678 N (single stand kneeling)*	*Quadriceps<br><br>(300 N)*	*Tekscan*

35. Huang et al. (2002)	*autograft, allograft*	*horizontal Rig*	*0°; 15°, 30°, 45°*	*N.A.*	*1200 N (axial)*	*N.A.*	*Fuji film*
36. Ihn et al. (1993)	*intact, partial (total) meniscectomy*	*material testing machine*	*0°*	*N.A.*	*3000 N (axial)*	*N.A.*	*Sensor*
37. Inaba et al. (1990)	*varus-valgus instability*	*experimental apparatus*	*0°*	*N.A*	*2700 N*	*N.A.*	*pressure transducers, Bourdon tube*
38. Kdolsky et al. (2004)	*uninjured knee pressure pattern → intraoperative pressure*	*flexed and hanging, as in a leg holder*	*N.A.*	*90°– 0 ° – 90 °*	*N.A.*	*N.A.*	*Tekscan*
39. Kenawey et al. (2011)	*intact, rotational alignment*	*specimen lying on table + foot plate*	*0° flex + 10°- 40° int./ext. rot.*	*N.A.*	*350 N (axial)*	*N.A.*	*Tekscan*
40. Kim et al. (2013)	*root tear, repair, total meniscectomy, allograft, MCL release*	*modified material testing machine*	*0°, 30°, 60°, 90°*	*N.A.*	*300 N(axial)*	*N.A.*	*Novel approach*
41. Koh et al. (2016)	*intact, horizontal cleavage tear, repair, leaf resection, resection of both leaves*	*desktop machine*	*0°, 60°*	*N.A.*	*800 N (axial)*	*N.A.*	*Tekscan*
42. Kurosawa et al. (1980)	*total meniscectomy*	*desktop machine*	*0°, 30°, 60°, 90°*	*N.A.*	*1500 N (axial)*	*N.A.*	*Silicone rubber*
43. LaPrade et al. (2015)	*root tear, anatomical (nonanatomical) transtibial pull-out repair*	*modified material testing machine*	*0°, 30°, 60°, 90°*	*N.A.*	*1000 N (axial)*	*N.A.*	*Tekscan*
44. Lee et al. (2006)	*radial tear, total meniscectomy*	*desktop machine*	*0°, 30°, 60°*	*N.A.*	*1800 N (axial)*	*N.A.*	*Tekscan*
45. Li et al. (2002)	*PCL deficiency*	*robotic machine*	*0–120°*	*N.A.*	*N.A.*	*Quadriceps 400 N Hamstring 200 N*	*N.A.*
46. Linder-Ganz et al. (2010)	*intact, total meniscectomy, implant*	*modified material testing machine*	*0°*	*N.A.*	*1200 N (axial)*	*N.A.*	*Tekscan*
47. Marchetti et al. (2017)	*intact, MCL tear and repair, bucket-handle tear, inside-out repair, all-inside repair*	*modified material testing machine*	*0°, 30°, 45°, 60°, 90°*	*N.A.*	*1000 N (axial)*	*N.A.*	*Tekscan*
48. Marzo et al. (2009)	*meniscal horn tear, repair*	*material testing machine*	*0°*	*N.A.*	*1800 N (axial)*	*N.A.*	*Tekscan*

This is a provisional file, not the final typeset article

49. McCulloch et al. (2016)	*intact, transplant, autograft*	*Oxford-rig*	*0°, 30°, 90°, 115°*	*flexion-extensi on cycle*	*267 N (axial – GRF)*	*Quadriceps (218 N), Hamstring (80 N)*	*Tekscan*
50. McDermott et al. (2008)	*intact, meniscectomy, allograft (bone block, suture)*	*modified material testing machine*	*0°*	*N.A.*	*700 N (axial)*	*N.A.*	*Fuji film*
51. MacDonald et al. (1996)	*posterior cruciate deficient knee*	*material testing system machine*	*0°, 30°, 60°*	*N.A.*	*1500 N*	*Quadriceps*	*Fuji Prescale film*
52. Meyer et al. (2008)	*test to failure, ACl rupture due to ex compressive loading*	*modified material testing machine*	*30°*	*N.A.*	*up to 5.5 kN*	*N.A.*	*Fuji Prescale film*
53. Mina et al. (2008)	*HTO for unloading osteochondral defect*	*material testing machine*	*30° flexion + 12° valgus to 10° varus (stepwise 2°).*	*N.A.*	*200 N*	*N.A.*	*Tekscan*
54. Muriuki et al. (2011)	*intact, tear, medial meniscal tear, repair, total meniscectomy*	*modified material testing machine*	*0°, 30°, 60°, 90°*	*N.A.*	*1000 N (axial)*	*N.A.*	*Fuji film*
55. Nakayama et al. (2005)	*diff. TKA designs by posterior force*	*parallel-link six-axis actuator*	*9°, 120°, 150°*	*N.A.*	*posterior load 500 N*	*N.A.*	*Tekscan*
56. Ode et al. (2012)	*intact, radial tear, repair, total meniscectomy*	*modified material testing machine*	*0°, 60°*	*N.A.*	*800 N (axial)*	*N.A.*	*Tekscan*
57. Ostermeier et al. (2006)	*TKA and tibiofemoral slope*	*knee simulator (isokinetic)*	*N.A.*	*120° – 0°*	*passive*	*Hamstring 200 N, Quadriceps 31 Nm*	*Tekscan*
58. Paci et al. (2009)	*intact, after release Anterior Intermeniscal Ligament*	*modified material testing machine*	*N.A.*	*0° to 60°*	*1000 N (axial)*	*N.A.*	*Tekscan*
59. Padalecki et al. (2014)	*root avulsion, repair, radial tear, in situ repair*	*modified material testing machine*	*0°, 30°, 45°, 60°, 90°*	*N.A.*	*1000 N (axial)*	*N.A.*	*Tekscan*
60. Paletta et al. (1997)	*intact, meniscectomy, allograft, release of anterior/posterior horn attachments of allograft*	*material testing machine*	*0°, 30°, 60°*	*N.A.*	*1800 N (axial)*	*N.A.*	*Fuji film*
61. Perez-Blanca et al. (2016)	*posterior root avulsion, repair, total meniscectomy*	*desktop machine*	*0°, 30°, 60°, 90°*	*N.A.*	*1000 N (axial)*	*N.A.*	*Tekscan*

62. Poh et al. (2012)	intact, after sectioning anterior intermeniscal ligament	modified material testing machine	0°	N.A.	1800 N (axial)	N.A.	Tekscan
63. Prince et al. (2014)	intact, lateral anterior horn tear, repair, meniscectomy	material testing machine	0°, 30°	N.A.	1000 N (axial)	N.A.	Tekscan
64. Rodner et al. (2006)	HTO, tibial slope	material testing machine	0°, 30°	N.A.	500 N	N.A.	Tekscan
65. Schall et al. (2019)	Proof-of-Concept: Novel Knee Joint Simulator	knee joint simulator, Oxford rig	N.A.	gait cycle, exercis es	GGF: over 800 N	Quadriceps Hamstring	Tekscan
66. Schillhammer et al. (2012)	posterior horn detachment, repair	modified material testing machine	N.A.	gait cycle	2000 N (axial), A-P force, rotation torque	N.A.	Tekscan
67. Seitz et al. (2012)	intact, partial (total) meniscectomy	modified material testing machine	0°, 30°, 60°	N.A.	1000 N (axial)	N.A.	Tekscan
68. Seitz et al. (2019)	open-wedge high tibial osteotomy (5°, 10°)	load-application system	0°, 30°	N.A.	1000 N (axial)	N.A.	Tekscan
69. Sekaran et al. (2002)	nonanatomic location (autolog. PCL)	load-application system	0°, 15°, 30°, 45°	N.A.	1200 N (axial)	N.A.	Fuji film
70. Shimakawa et al. (2019)	intact, ACL reconstruction, partial- + subtotal meniscectomy	load-application system	0°, 30°, 60°, 90°	N.A.	735 N	N.A.	Tekscan
71. Shiramizu et al. (2009)	high flexion knee designs	mechanical testing machine	0, 30, 60, 90, 110, 135, 155°	N.A.	3600 N	N.A.	Tekscan
72. Stein et al. (2019)	partial medial meniscectomy, partial meniscal replacement	mechanical testing machine	N.A.	flexion cycle, squat, 0° to 100°	200 N	N.A.	Tekscan
73. Steinbruck et al. (2016)	TKA posterior-stabilized vs. medial-stabilized design	Oxford rig	N.A	active deep knee flexion from 20° to 120°	N.A.	Quadriceps Hamstring	Tekscan

This is a provisional file, not the final typeset article

74. Stukenborg-Colsman et al. (2000)	*intact, before and after total knee arthroplasty (TKA)*	*knee simulator (horizontal)*	*N.A*	*120° – 0°*	*N.A.*	*Quadriceps , 31 Nm ext. moment*	*Tekscan*
75. Stukenborg-Colsman et al. (2002)	*intact, before and after total knee arthroplasty (TKA)*	*knee simulator (horizontal)*	*N.A*	*120° – 0°*	*N.A.*	*Quadriceps , 31 Nm ext. moment*	*Tekscan*
76. Thambya et al. (2007)	*contact stresses at both compartments*	*6-DOF holding apparatus*	*15.5° flexion and slight varus angle 2°*	*N.A.*	*1144 N*	*N.A.*	*Tekscan*
77. Uquillas et al. (2017)	*intact, horizontal cleavage tear, flap removal*	*desktop machine*	*N.A.*	*−5° to 135°*	*500 N (axial) 100 N P shear 2.5 Nm torque*	*Quadriceps*	*Tekscan*
78. Van Egmond et al. (2017)	*HTO, MCL release*	*test setup*	*0°*	*N.A.*	*N.A.*	*N.A.*	*Tekscan*
79. Van Thiel et al. (2011)	*total meniscectomy, transplant*	*material testing machine*	*0°*	*N.A.*	*800 N (axial)*	*N.A.*	*Tekscan*
80. Verma et al. (2008)	*meniscectomy, transplant*	*material testing machine*	*0°, 30°*	*N.A.*	*1000 N (axial)*	*N.A.*	*Tekscan*
81. Vrancken et al. (2016)	*intact, implant, total meniscectomy, allograft*	*Horizontal Rig*	*N.A.*	*0° to 90°*	*1000 N (axial), 76 N (A-P force) 3.4 Nm (rotation torque)*	*Quadricep (190 N), RF (250 N)*	*Tekscan*
82. Walker et al. (1975)	*intact*	*test setup*	*0°, 30°, 60°, 90°*	*N.A.*	*0 N, 1000 N, 1500 N*	*N.A.*	*Miniature contact pressure transducer*
83. Walker et al. (2015)	*intact*	*Horizontal Rig*	*N.A.*	*−5° to 135°*	*500 N (axial) + 100 N a.-p. shear*	*N.A.*	*Tekscan*
84. Wang et al. (2015)	*intact, meniscectomy, autograft transplantation*	*modified load-controlled knee simulator*	*N.A.*	*gait cycle*	*N.A.*	*N.A.*	*Tekscan*
85. Willinger et al. (2019)	*neutral, varus/valgus malalignment*	*modified material testing machine*	*0° flexion 10%, 20% varus/valgus*	*N.A.*	*750 N*	*N.A.*	*Tekscan*

86. Willinger et al. (2020)	*sequential medial meniscus resection, intersect the tibia plateau at 30%, 40%, 50%, 60%, 70%*	*universal testing machine*	*0°*	*N.A.*	*750 N*	*N.A.*	*Tekscan*
87. Yazdi et al. (2014)	*intact, tibial rotation*	*lower limb loading apparatus*	*0°*	*N.A.*	*Half BW of each specimen*	*N.A.*	*Fuji film*
88. Yazdi et al. (2016)	*intact, partial fibulectomy*	*lower limb loading apparatus*	*0°*	*N.A.*	*Half BW of each specimen*	*N.A.*	*Fuji film*
89. Zhang et al. (2015)	*intact, repair, meniscectomy*	*modified material testing machine*	*0°, 8°, 15°, 30°*	*N.A.*	*250 N, 500 N, 1000 N*	*N.A.*	*Tekscan*

This is a provisional file, not the final typeset article
